# Angiotensin II receptor blocker intake associates with reduced markers of inflammatory activation and decreased mortality in patients with cardiovascular comorbidities and COVID-19 disease

**DOI:** 10.1371/journal.pone.0258684

**Published:** 2021-10-21

**Authors:** Sebastian Cremer, Lisa Pilgram, Alexander Berkowitsch, Melanie Stecher, Siegbert Rieg, Mariana Shumliakivska, Denisa Bojkova, Julian Uwe Gabriel Wagner, Galip Servet Aslan, Christoph Spinner, Guillermo Luxán, Frank Hanses, Sebastian Dolff, Christiane Piepel, Clemens Ruppert, Andreas Guenther, Maria Madeleine Rüthrich, Jörg Janne Vehreschild, Kai Wille, Martina Haselberger, Hanno Heuzeroth, Arne Hansen, Thomas Eschenhagen, Jindrich Cinatl, Sandra Ciesek, Stefanie Dimmeler, Stefan Borgmann, Andreas Zeiher, on behalf of the LEOSS study group

**Affiliations:** 1 Department of Medicine, Cardiology, Goethe University Hospital, Frankfurt, Germany; 2 German Center for Cardiovascular Research DZHK, Partner Site Rhine-Main, Berlin, Germany; 3 Cardiopulmonary Institute, Goethe University Frankfurt, Frankfurt, Germany; 4 Department of Internal Medicine, Hematology/Oncology, Goethe University Frankfurt, Frankfurt am Main, Germany; 5 Department I for Internal Medicine, Faculty of Medicine and University Hospital Cologne, University of Cologne, Cologne, Germany; 6 Internal Medicine II, Department of Infectious Diseases, Freiburg University Hospital, Freiburg, Germany; 7 Institute for Cardiovascular Regeneration, Goethe University Frankfurt, Frankfurt, Germany; 8 Institute of Medical Virology, University of Frankfurt, Frankfurt, Germany; 9 Department of Internal Medicine II, Technical University of Munich, Hospital rechts der Isar, Munich, Germany; 10 University Hospital Regensburg, Regensburg, Germany; 11 Department of Infectious Diseases, University Hospital Essen, Essen, Germany; 12 Department of Internal Medicine I, Hospital Bremen-Mitte, Bremen, Germany; 13 Department of Internal Medicine II, Giessen University, Giessen, Germany; 14 Department of Internal Medicine II, Jena University, Jena, Germany; 15 University Clinic for Hematology, Oncology, Hemostaseology and Palliative Care, University of Bochum, Minden, Germany; 16 Department of Internal Medicine I, Hospital Passau, Passau, Germany; 17 Department of Emergency and Intensive Care Medicine, Klinikum Ernst von Bergmann, Potsdam, Germany; 18 Department of Experimental Pharmacology and Toxicology, University Medical Center Hamburg-Eppendorf, Hamburg, Germany; 19 Department of Infectious Diseases and Infection Control, Ingolstadt Hospital, Ingolstadt, Germany; Osaka University Graduate School of Medicine, JAPAN

## Abstract

**Aims:**

Patients with cardiovascular comorbidities have a significantly increased risk for a critical course of COVID-19. As the SARS-CoV2 virus enters cells via the angiotensin-converting enzyme receptor II (ACE2), drugs which interact with the renin angiotensin aldosterone system (RAAS) were suspected to influence disease severity.

**Methods and results:**

We analyzed 1946 consecutive patients with cardiovascular comorbidities or hypertension enrolled in one of the largest European COVID-19 registries, the Lean European Open Survey on SARS-CoV-2 (LEOSS) registry. Here, we show that angiotensin II receptor blocker intake is associated with decreased mortality in patients with COVID-19 [OR 0.75 (95% CI 0,59–0.96; p = 0.013)]. This effect was mainly driven by patients, who presented in an early phase of COVID-19 at baseline [OR 0,64 (95% CI 0,43–0,96; p = 0.029)]. Kaplan-Meier analysis revealed a significantly lower incidence of death in patients on an angiotensin receptor blocker (ARB) (n = 33/318;10,4%) compared to patients using an angiotensin-converting enzyme inhibitor (ACEi) (n = 60/348;17,2%) or patients who received neither an ACE-inhibitor nor an ARB at baseline in the uncomplicated phase (n = 90/466; 19,3%; p<0.034). Patients taking an ARB were significantly less frequently reaching the mortality predicting threshold for leukocytes (p<0.001), neutrophils (p = 0.002) and the inflammatory markers CRP (p = 0.021), procalcitonin (p = 0.001) and IL-6 (p = 0.049). ACE2 expression levels in human lung samples were not altered in patients taking RAAS modulators.

**Conclusion:**

These data suggest a beneficial effect of ARBs on disease severity in patients with cardiovascular comorbidities and COVID-19, which is linked to dampened systemic inflammatory activity.

## Introduction

The corona virus disease 2019 (COVID-19) pandemic imposes a significant burden on health care systems [1]. COVID-19 progression is characterized by three distinct phases: an initial infection phase followed by a respiratory distress phase and finally culminating in a severe hyperinflammatory state [2, 3]. Patients with comorbid conditions including hypertension and cardiovascular diseases are highly represented among patients, who suffer from a critical course of COVID-19 and eventually succumb to the disease, indicating that these comorbidities may predispose to increased aggressiveness of the infection in this group [4, 5]. Importantly, patients with cardiovascular comorbidities display an exaggerated inflammatory response and increased thrombogenic activation already in the early stage of the disease [6].

The Severe Acute Respiratory Distress Syndrome Coronavirus 2 (SARS-CoV-2) enters the cell by binding of its trimeric spike protein to the human receptor angiotensin-converting enzyme II (ACE2) expressed on epithelial cells in the respiratory system [7]. Thus, drugs which regulate ACE2 expression, were considered as potential mediators of the increased risk for COVID-19 patients with cardiovascular comorbidities [8]. Angiotensin-converting enzyme inhibitors (ACEi) and angiotensin II receptor blockers (ARBs) are commonly used disease modifying drugs in hypertension and most cardiovascular diseases. Animal studies showed that ACEIs and ARBs can upregulate ACE-2 [9], which raised concerns whether a higher availability of the entry receptor for SARS-CoV2 might result in increased infection rates and higher disease severity in patients with RAAS modulating drugs. However, association of ACEi/ARB use with outcomes in patients with COVID-19 revealed conflicting results with respect to disease progression with most studies reporting neutral or even beneficial effects of blockade of the renin angiotensin aldosterone system (RAAS) [10–12].

Therefore, it was the aim of the present study to analyze the effect of ARB/ACEi use specifically in patients with preexisting cardiovascular disease or hypertension after SARS-CoV2 infection. Data from one of the largest European COVID-19 registries, the Lean European Open Survey on SARS-CoV-2 Infected Patients (LEOSS) registry (www.leoss.net) was analyzed.

## Material and methods

### Study population

The study population consists of 1946 consecutive patients with documented cardiovascular comorbidity and/or hypertension, who were included in the LEOSS registry between 03/18/2020 and 10/14/2020 across 100 hospitals in Europe, most of them in Germany. All patients had a SARS-CoV-2 infection confirmed by positive results of PCR testing. Data collection was performed retrospectively and anonymously. Patients were stratified into the following groups after an initial positive test result for SARS-CoV-2: patients in the *uncomplicated phase* were either asymptomatic, had symptoms of upper respiratory tract infection, fever or nausea, emesis or diarrhea. Patients in the *complicated phase* had at least one of the following characteristics: new need for oxygen supplementation or clinically relevant increase of prior oxygen home therapy, PaO2 at room air < 70 mmHg, SO2 at room air < 90%, increase of AST or ALT > 5x ULN (upper limit of normal), new cardiac arrythmia, new pericardial effusion > 1cm or new heart failure with pulmonary edema, congestive hepatopathy or peripheral edema. Patients in the *critical phase* were dependent on catecholamines, experienced life-threatening cardiac arrhythmia, had mechanical ventilation (invasive or non-invasive) or need for unplanned mechanical ventilation prolongation (>24h) of planned mechanical ventilation, liver failure with an INR >3.5 (quick <50%), a qSOFA score of > = 2 or acute renal failure with need of dialysis. Approval for LEOSS was obtained by the applicable local ethics committees of all participating centers and registered at the German Clinical Trails Register (DRKS, No. S00021145). As all data are based on anonymous reports, written informed consent was waived by the local ethics committees.

### Data collection

Demographic, clinical, laboratory, treatment, and outcome data were extracted from medical records. Analyzed laboratory data were collected within 48 hours of a positive PCR result irrespective of the patient’s status. Therefore, all biomarker measurements represent baseline values. Data were distributed into different categories. Cardiovascular (CV) comorbidity was defined by one of the following diseases: coronary artery disease, prior myocardial infarction, chronic heart failure, atrial fibrillation, AV-Block, aortic valve stenosis, peripheral artery disease, carotid artery disease or cerebrovascular disease. Included were those patients, for which information about RAAS medication was available. Several steps are taken to prevent re-identification, which include vertical (categorical assessment of numerical variables) and horizontal data aggregation (data aggregation within the phases of disease). In order to ensure anonymity in all steps of the analysis process, an individual LEOSS Scientific Use File (SUF) was created, which is based on the LEOSS Public Use File (PUF) principles described in Jakob et al. [13].

### Human lung samples

In addition to the LEOSS data, 29 primary lung samples were obtained from patients undergoing lung surgery for cancer at the University Hospital Giessen. 15 of those patients were not treated with RAAS modulators, 10 were on chronic ACEi therapy and four patients on ARB treatment. Chronic therapy was defined as treatment duration for at least three months. All patients gave informed consent. This study was approved by the local ethics committee of the Justus-Liebig-University Gießen.

Prior to immunostaining, paraffin was removed from 4μm thick histological sections by incubating them 30 min at 60°C. Then, the histological sections were rehydrated in a series of ethanol washes. Antigen retrieval was performed in citrate buffer, 10mM pH 6.0, for 90 secs in a steam pressure. Samples were then cooled down by rising with tab water. Tissue was permeabilized with 3 consecutive 5 min washes with PBS/0.1%Triton X-100 and blocked for one hour in blocking solution (1% BSA, 2% donkey serum in PBS). Primary antibodies were incubated overnight at 4°C in blocking solution. The consecutive day, 3 consecutive 5 min washes of PBS/0.1% Triton X-100 were performed to wash the primary antibodies. Secondary antibodies were incubated in PBS/0.1% Triton X-100 for 1 hour at room temperature, washed 3 times for 5 min with PBS/0.1% Triton X-100 and mounted with mounting media containing Hoechst. Immunostainings were imaged in a Leica SP8 confocal inverted microscope and quantified using Volocity image analysis software 6.5.1 (Quorum Technologies).

Primary antibodies: Goat anti-ACE2 (AF933, R&D) 1:50, Biotinylated Ulex Europaeus Agglutinin I (B1065, Vector). Secondary antibodies: Donkey anti-goat Alexa Fluor-555 (A21432, Invitrogen) and Streptavidin-647 (S32357, Life Technologies).

### Statistical analysis

All data are presented as categorical variables (values and proportions) and were analyzed with χ^2^ or Fisher exact test, where appropriate. Outcome in patients was analyzed with Cox- hazard regression analysis and parameters associated with outcome were further included in multivariate analysis for identification of independent predictors. The multivariate analysis was performed using logistic regression according to Wald approach in step-down mode. Primary endpoint of the study was death of any cause.

For survival analysis within the individual groups, Kaplan–Meier analyses were used and 95% confidence intervals (CI) were calculated. Log-rank testing was applied for comparison of event-free survival analysis. Cut-offs for biomarkers were optimized by maximizing sensitivity plus specificity from a receiver operating characteristic (ROC) curve analysis from censored data with a nearest neighbor estimation with smoothing parameter as small as possible to ensure monotone ROCcurves [14].

For all statistical analyses, *p* <0.05 was considered to be significant. Analyses were performed with SPSS, version 26 (IBM, Chicago, Illinois).

## Results

### Characteristics of patients at baseline

Baseline demographics of all included patients irrespective of disease phase are illustrated in Table 1. Among the 1946 patients included in the study, 854 patients did not take RAAS-inhibitors, whereas 599 patients were on ACE-inhibitors and 493 patients were taking an ARB. Patients with an ARB where more often in an uncomplicated phase of the disease at baseline (p<0.001). There were no significant differences with respect to overall cardiovascular comorbidity between the three groups of patients (p = 0.337), however, coronary artery disease (p = 0.009) and a history of myocardial infarction (p = 0.001) was more prevalent in patients taking an ACE-inhibitor. Patients with an RAAS-inhibitor were more likely to have hypertension and diabetes mellitus compared to patients who did not take an ARB or an ACE-inhibitor (p<0.001 and p = 0.005). In addition, chronic kidney disease was more prevalent in patients taking an ARB (p = 0.028), and statin intake was more prevalent in both groups, patients with an ACE-inhibitor and taking an ARB (p<0.001; Table 1).

**Table 1:** Baseline characteristics of patients included in the study.

	No RAAS Inhibition	ACEi	ARB	P-value
	n = 854	n = 599	n = 493	
**Demographics**				
Age, years				
< 25 years	0	0	0	0.001
26–35 years	13 (1,52%)	3 (0,50%)	1 (0,20%)	
36–45 years	30 (3,51%)	19 (3,17%)	4 (0,81%)	
46–55 years	99 (11,59%)	47 (7,84%)	40 (8,11%)	
56–65 years	162 (18,96%)	105 (12,29%)	111 (22,51%)	
66–75 years	190 (22,24%)	131 (21,86%)	100 (20,28%)	
76–85 years	251 (29,39%)	200 (33,38%)	184 (37,32%)	
> 85 years	107 (12,53%)	92 (15,36%)	52 (10,55%)	
Sex (male), n (%)	525 (61,47%)	381 (63,61%)	153 (57,20%)	0.092
Uncomplicated Stage	466 (54,56%)	348 (58,09%)	318 (64,5%)	<0.001
Complicated Stage	273 (31,97%)	204 (34,06%)	143 (29,01%)	
Critical Stage	115 (13,47%)	47 (7,85%)	32 (6,49%)	
**Clinical History**				
Cardiovascular Comorbidity	509 (59,60%)	369 (61,6%)	282 (57,2%)	0.337
Coronary Artery Disease	206 (24,12%)	188 (31,38%)	133 (26,98%)	0.009
History of MI	75 (8,78%)	91 (15.19%)	48 (9.73%)	0.001
Chronic Heart Failure	300 (35,12%)	216 (35,06%)	164 (33,26%)	0.628
Aortic Valve Stenosis	38 (3,74%)	23 (3,83%)	25 (5,07%)	0.58
Atrial Fibrillation	211 (24,71%)	149 (24,87%)	99 (20,08%)	0.105
AV-Block	32 (3,75%)	25 (4,17%)	19 (3,85%)	0.922
Carotid artery disease	37 (4,33%)	28 (4,67%)	27 (5,47%)	0.624
Peripheral artery disease	79 (9,25%)	39 (6,51%)	33 (6,69%)	0.098
Cerebral vascular disease	112 (13,11%)	100 (16,69%)	68 (13,79%)	0.174
Hypertension	693 (81,14%)	548 (91,48%)	457 (92,70%)	p<0.001
Diabetes	230 (26,93%)	208 (34,72%)	158 (32,04%)	0.005
Chronic kidney disease	227 (26,58%)	143 (24,87%)	76 (31,03%)	0.028
Chronic pulmonary disease	154 (18,03%)	85 (14,19%)	92 (20,21%)	0.084
Cancer	138 (16,16%)	99 (16,53%)	87 (17,65%)	0.776
Statin Treatment	854 (22,13%)	235 (39,23%)	198 (40,16%)	p<0.001

#### Outcomes and predictors

**Fig 1:**
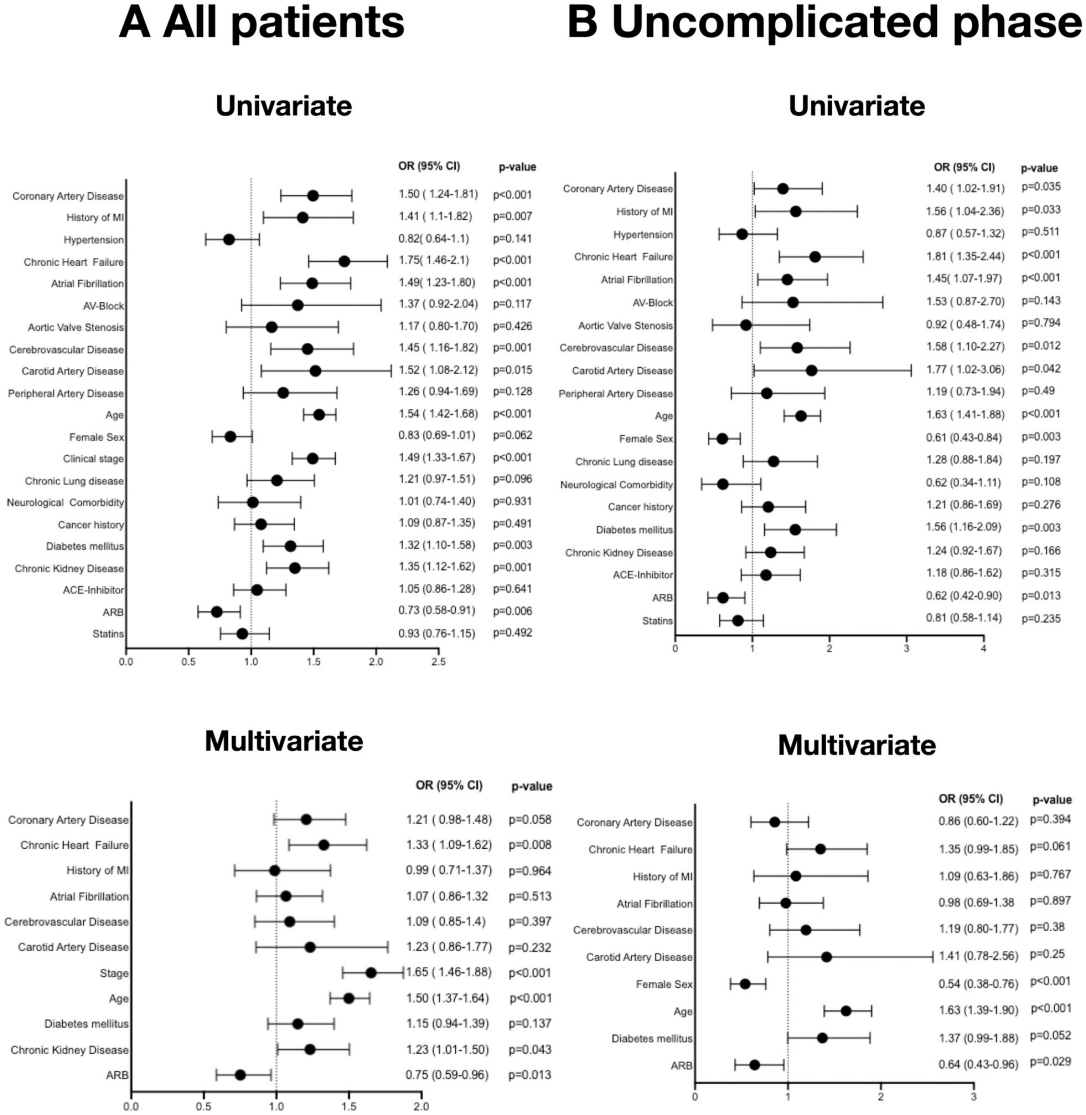
Mortality risk in patients with CVD and hypertension. Forrest plots of univariate and multivariate analysis for mortality risk for the given clinical characteristics of all included patients (**A**) and patients who presented in the uncomplicated phase at baseline (n = 1132) (**B)**.

Mortality risk of all patients was calculated with Cox-hazard regression analysis. Here, increasing age [HR 1.54 (95% CI 1.42–1.68; p<0.001)] and advanced clinical stage [HR 1.49 (95% CI 1.33–1.67; p<0.001)] were associated with an increased risk of death after infection with SARS-CoV2. In addition, there was a significantly higher mortality risk when patients had diabetes mellitus [HR 1.32 (95% CI 1.1–1.58; p = 0.003)] or chronic kidney disease [HR 1.35 (95% CI 1.12–1.62; p = 0.001)]. Moreover, cardiovascular conditions including coronary artery disease [HR 1.50 (95% CI 1.24–1.81; p<0.001)], previous myocardial infarction [HR 1.41 (95% CI 1.1–1.82; p = 0.007)], chronic heart failure [OR 1.75 (95% CI 1.46–2.1; p<0.001)], atrial fibrillation [HR 1.49 (95% CI 1.23–1.80; p<0.001)], cerebrovascular disease [HR 1.45 (95% CI 1.16–1.82; p = 0.001)] as well as carotid artery disease [HR 1.52 (95% CI 1.08–2.12; p = 0.015)] were associated with mortality risk in univariate analysis. Interestingly, ARB medication was associated with a lower risk of mortality [HR 0.73 (95% CI 0.58–0.91; p = 0.006)], whereas ACE-inhibitors had no influence on prognosis (Fig 1A).

To define independent predictors of death, multivariate analysis was performed. Here, patient age [HR 1.5 (95% CI 1.37–1.64; p<0.001)] and clinical stage at baseline [HR 1.65 (95% CI 1.46–1.88; p<0.001)] remained independent predictors of death. Among preexisting medical conditions, only chronic kidney disease [HR 1.23 (95% CI 1.01–1.5; p = 0.043)] and chronic heart failure [HR 1.33 (95% CI 1.09–1.62; p = 0.008)] were predictive for mortality. Again, ARB intake was independently associated with a lower risk of death in all patients with SARS-Cov2-Infection, irrespective of disease stage at baseline in multivariate analysis [HR 0.75 (95% CI 0.59–0.96; p = 0.013)] (Fig 1A).

To further dissect a potential influence of clinical stage at baseline on the protective effects of ARB intake in patients with COVID-19, we stratified the patients according to disease stage at diagnosis and applied a similar analysis. Among the patients in the uncomplicated phase at baseline in univariate analysis, patient age [HR 1.63 (95% CI 1.41–1.88; p<0.001)] and female gender [HR 0.61 (95% CI 0.43–0.84; p = 0.003)] emerged as predictors of mortality. In addition, diabetes mellitus retained its prognostic value for mortality [HR 1.56 (95% CI 1.16–2.09; p = 0.003)], as did coronary artery disease [HR 1.4 (95% CI 1.02–1.91; p = 0.035)], a previous MI [HR 1.56 (95% CI 1.04–2.36; p = 0.033)], chronic heart failure [HR 1.81 (95% CI 1.35–2.44; p<0.001)], atrial fibrillation [HR 1,45 (95% CI 1,07–1,97; p<0.001)], cerebrovascular disease [HR 1.58 (95% CI 1.1–2.27; p = 0.012)] and carotid artery disease [HR 1.77 (95% CI 1.02–3.06; p = 0.042)]. Moreover, ARB prevented mortality in this patient cohort [HR 0.62 (95% CI 0.42–0.90; p = 0.013) Fig 1B]. Multivariate analysis revealed that age [HR 1.63 (95% CI 1.39–1.90; p<0.001)] and female gender [HR 0.54 (95% CI 0.38–0.76; p<0.001)] were the only independent predictors of mortality, whereas ARB intake was associated with a reduced risk for mortality after COVID-19 infection [HR 0.64 (95% CI 0.43–0.96; p = 0.029), Fig 1B].

**Fig 2:**
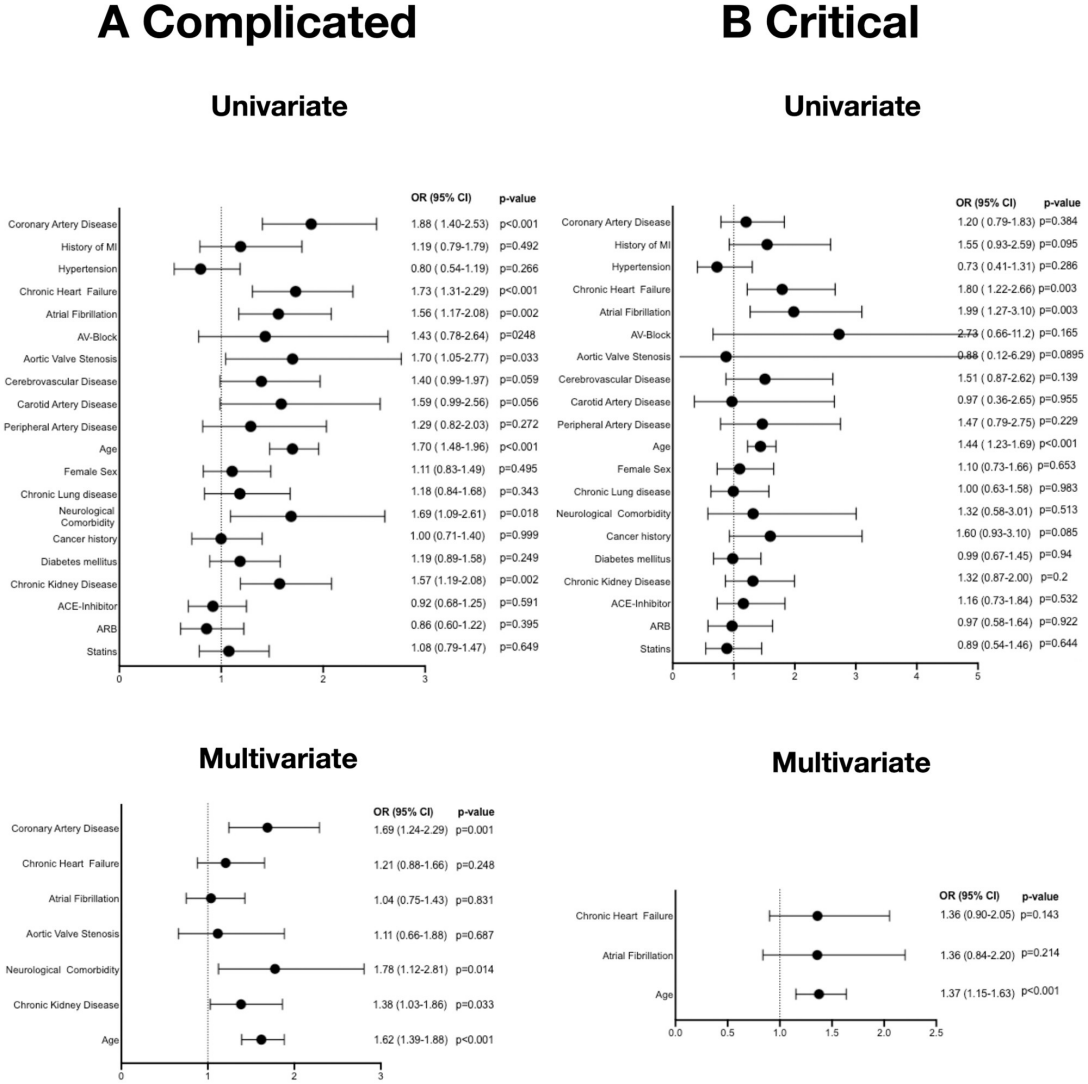
Mortality risk in patients with CVD and hypertension—Advanced disease. Forrest plots of univariate and multivariate analysis for mortality risk for the given clinical characteristics of patients who presented in the complicated phase (n = 620) **(A)** and those who presented in the critical phase (n = 194) **(B)**.

However, in the complicated phase, the presence of coronary artery disease, preexisting neurological comorbidities, chronic kidney disease and higher age were independently associated with an increased risk of death, while neither ARB nor ACE inhibitor intake did have an influence on prognosis in multivariate analysis (Fig 2A).

Lastly, in patients who initially presented in the critical phase of COVID-19 only higher age remained independently predictive after multivariate adjustment [HR 1.37 (95% CI 1.15–1.63; p<0.001; Fig 2B].

#### ARB intake is associated with improved prognosis in COVID-19 disease

**Fig 3:**
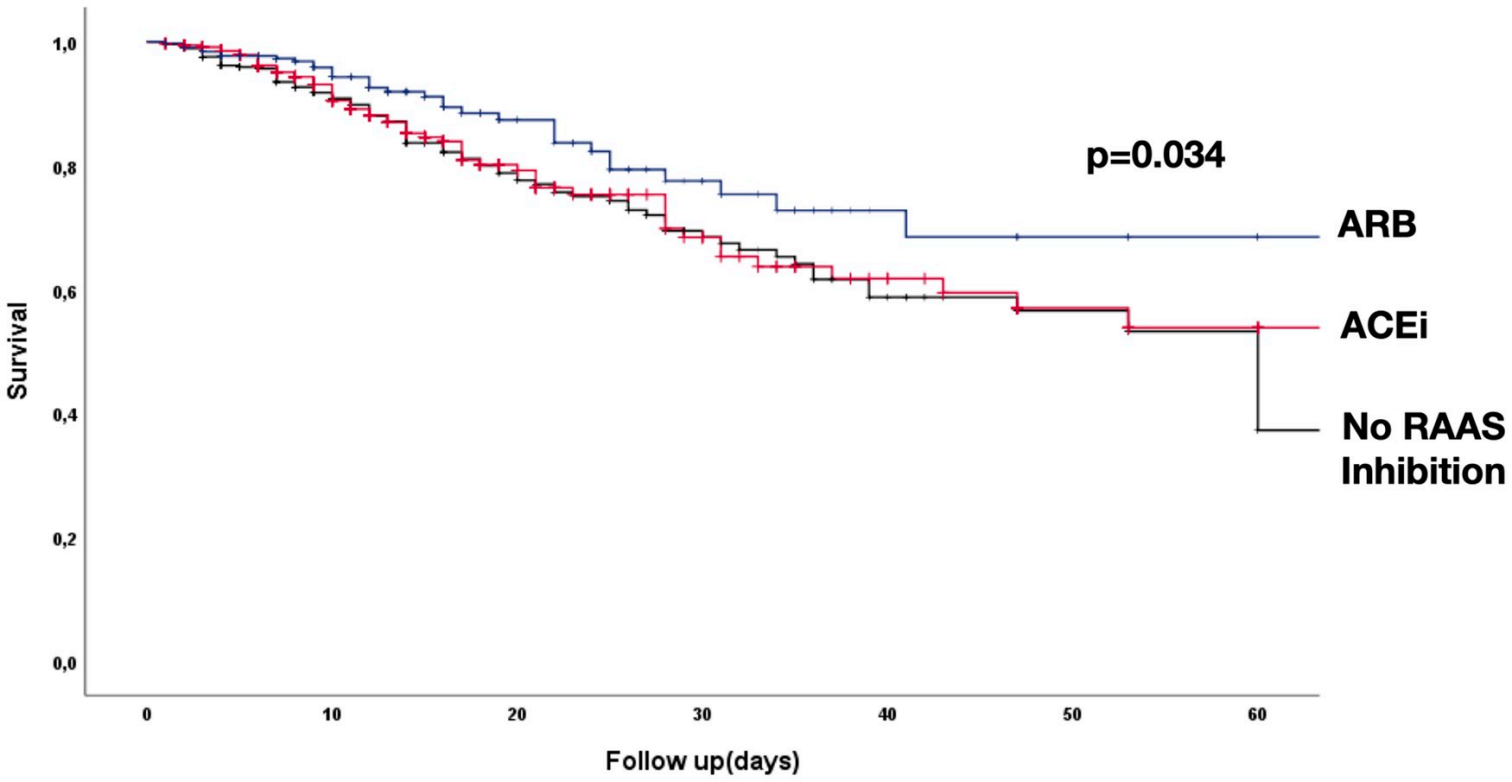
Kaplan-Meier analysis, patients with CVD and/or hypertension, baseline uncomplicated. Association between intake of RAAS- modulating drugs and mortality by Kaplan–Meier analysis in patients in the uncomplicated phase. *P*-value is for log-rank test, n = 1132.

This data indicate that the intake of ARB exerts its protective effect predominantly in patients, who were admitted in an uncomplicated phase of COVID-19. Indeed, Kaplan-Meier analysis revealed a significantly lower incidence of death in patients on an ARB (10,4%) compared to patients using an ACEi (17,2%) or patients who did receive neither an ACEi nor an ARB at baseline in the uncomplicated phase (19,3%; p<0.034; Fig 3). Importantly, baseline characteristics of patients with an ARB were not significantly different with respect to age, sex, and total burden of cardiovascular comorbidities, whereas patients taking an ACEi were more likely to have coronary artery disease or a history of myocardial infarction (Table 2).

**Table 2:** Baseline characteristics of patients in the uncomplicated phase of COVID-19.

	No RAAS Inhibition	ACEi	ARB	P-value (ARB vs No ARB)
	n = 466	n = 348	n = 318	
**Demographics**				
Age, years				
< 25 years	2 (0,43%)	0	0	0,370
26–35 years	10 (2,15%)	2 (0,57%)	0	
36–45 years	23 (4,94%)	14 (4,02%)	3 (0,94%)	
46–55 years	50 (10,73%)	32 (9,2%)	24 (7,55%)	
56–65 years	86 (18,45%)	69 (19,83%)	78 (24,53%)	
66–75 years	96 (20,6%)	74 (21,26%)	58 (18,24%)	
76–85 years	142 (30,47%)	114 (32,76%)	123 (38,68%)	
> 85 years	57 (12,23%)	43 (12,36%)	32 (10,06%)	
Sex (male), n (%)	277 (59,44%)	218 (62,64%)	177 (55,66%)	0.07
**Clinical History**				
Cardiovascular Comorbidity	280 (60,09%)	208 (59,77%)	177 (55,66%)	0.307
Coronary Artery Disease	115 (24,68%)	110 (31,61%)	76 (23,90%)	0.019
History of MI	38 (8,15%)	52 (14,94%)	23 (7.23%)	0.002
Chronic Heart Failure	160 (34,33%)	127 (36,49%)	99 (31,13%)	0.163
Aortic Valve Stenosis	21 (4,15%)	14 (4,02%)	14 (4,40%)	0.848
Atrial Fibrillation	113 (24,25%)	74 (21,26%)	69 (21,7%)	1,000
AV-Block	20 (4,29%)	16 (4,6%)	15 (4,72%)	1,000
Carotid artery disease	18 (3,86%)	12 (3,45%)	16 (5,03%)	0.34
Peripheral artery disease	40 (8,58%)	21 (6,03%)	21 (6,6%)	0.751
Cerebral vascular disease	60 (12,88%)	53 (15,23%)	44 (13,84%)	0.661
Hypertension	367 (78,76%)	319 (91,67%)	297 (93,40%)	0.462
Diabetes	117 (25.11%)	107 (30,75%)	100 (31,45%)	0.867
Chronic kidney disease	123 (26,39%)	70 (20.11%)	87 (27,36%)	0.029
Chronic pulmonary disease	64 (13,73%)	50 (14,37%)	58 (18,24%)	0.207
Cancer	90 (19,31%)	59 (16,95%)	51 (16,04%)	0.755
Statin Treatment	108 (23,18%)	131 (37,64%)	128 (40,25%)	0.315

#### Mitigation of cardiac damage and thrombo-inflammatory responses in patients with ARB intake

**Fig 4:**
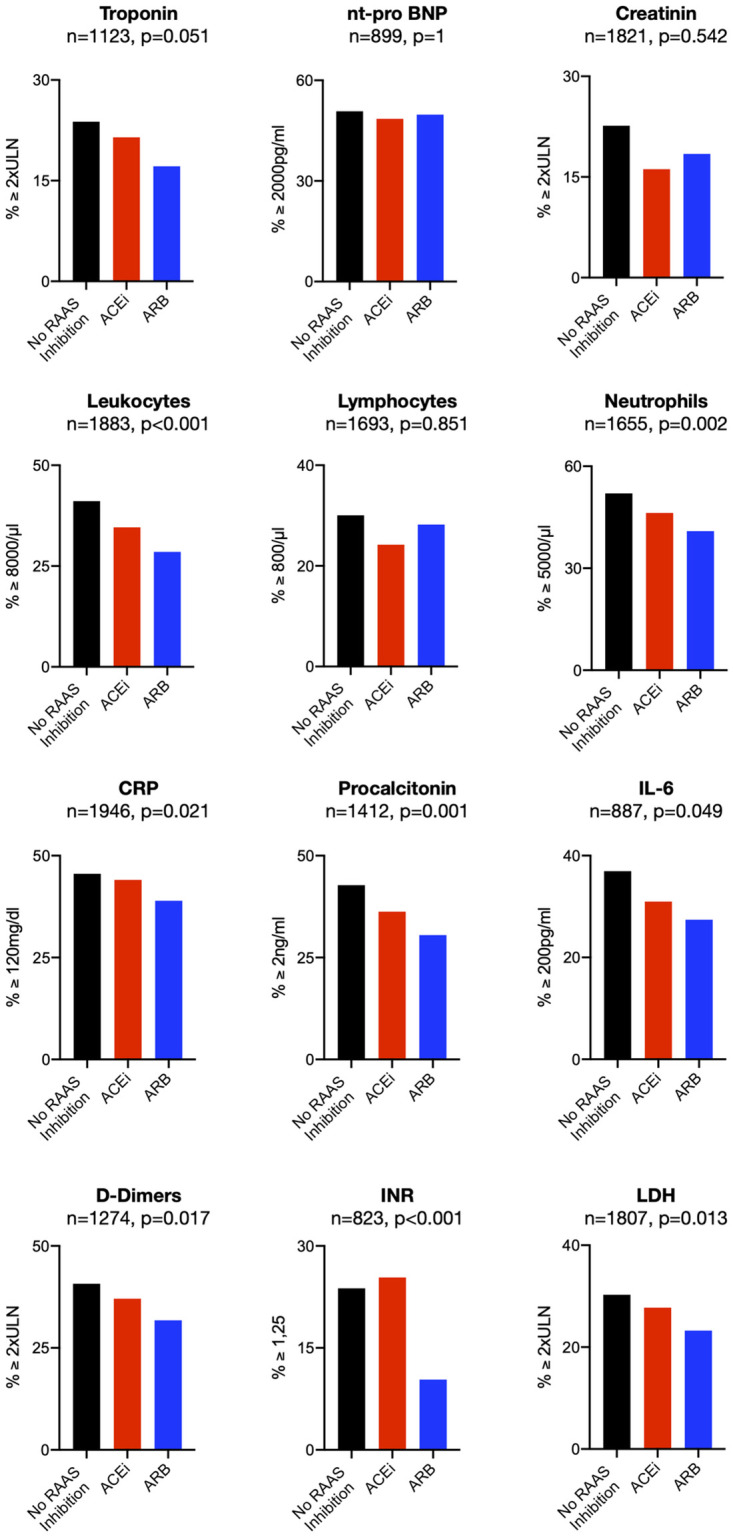
Biomarkers in all patients with CVD/Hypertension. Percentage of patients above the mortality predicting threshold for biomarkers calculated by ROC analysis in all patients with cardiovascular disease or hypertension irrespective of disease phase at baseline for patients without RAAS-modulation, patients on ACE-inhibitors and patients taking ARBs. n-numbers and p-values are depicted above each panel.

After having established ARB intake as an independent factor associated with reduced mortality in patients with COVID-19 disease and accompanying cardiovascular comorbidities or hypertension, we sought to explore potential mechanisms. Therefore, we analyzed biomarker levels for cardiac damage, activation of the coagulation system and inflammation in patients on ARB compared to patients taking ACEi or no RAAS-inhibition. In order to identify optimal cut-off points for biomarker levels to predict clinical outcome individually, we calculated survival receiver operating characteristics (survivalROC) curves for the single clinical endpoint all-cause mortality. Next, we assessed the proportion of patients with biomarker levels above these cutoffs. Here, we observed a strong trend towards lower troponin levels in patients taking an ARB (p = 0.051). Moreover, a significantly lower number of patients taking an ARB reached the mortality predicting threshold for leukocytes (p<0.001), neutrophils (p = 0.002) and the inflammatory markers CRP (p = 0.021), procalcitonin (p = 0.001) and IL-6 (p = 0.049). Also, D-dimers (p = 0.017) and INR (p<0.001) were less frequently elevated, indicating lower levels of thrombogenic activation in patients with an ARB. In addition, LDH levels were lower in those patients, too (p = 0.013; Fig 4).

**Fig 5:**
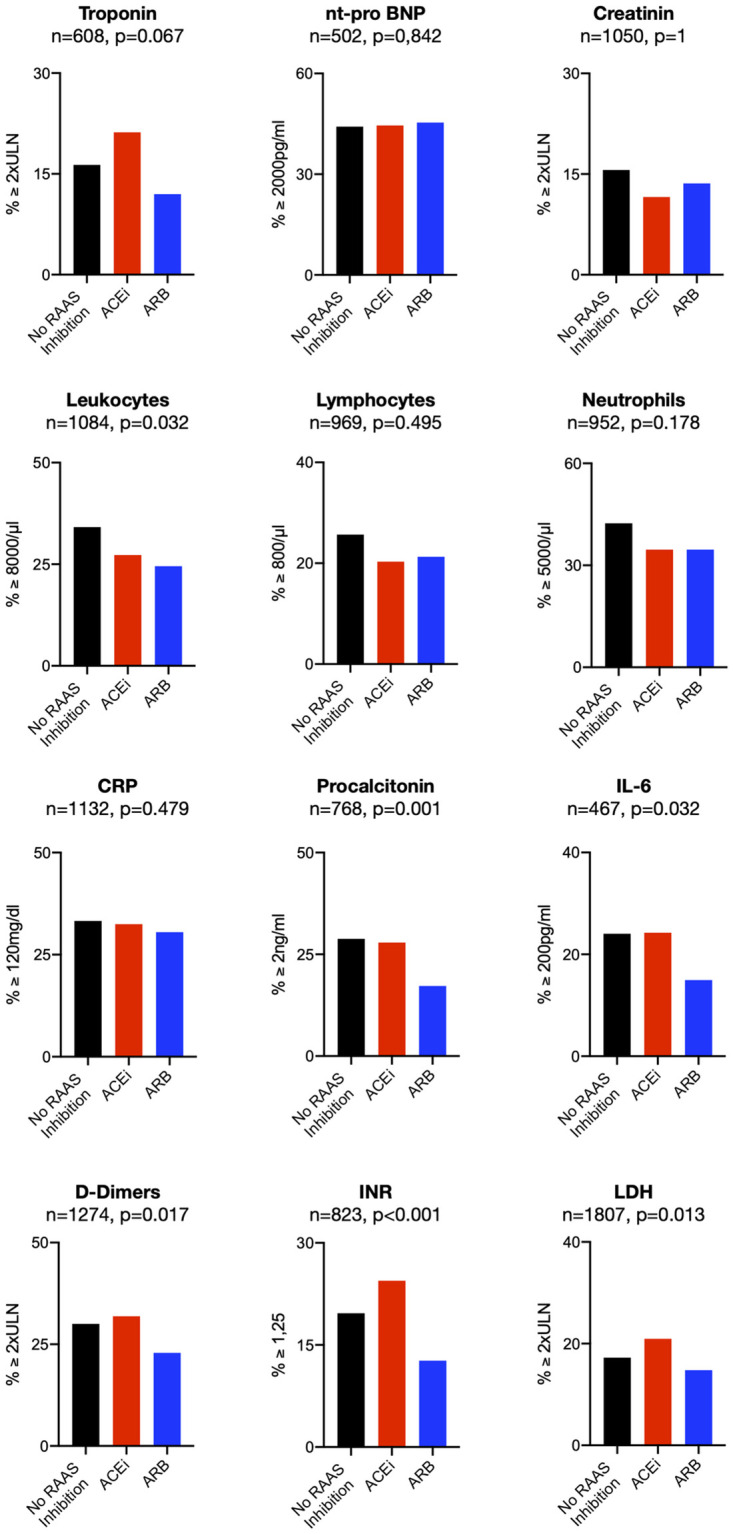
Biomarkers in uncomplicated patients with CVD/Hypertension. Percentage of patients above the mortality predicting threshold for biomarkers calculated by ROC analysis in patients in an uncomplicated stage of COVID-19 at baseline with cardiovascular disease for patients without RAAS-modulation, patients on ACEi and patients taking ARBs. n-numbers and p-values are depicted above each panel.

Because the protective effect on mortality was primarily driven by patients who presented in the uncomplicated phase, biomarker level analysis was extended to this subgroup of patients. Here, we confirmed a trend towards less cardiac damage as assessed by troponin levels (p = 0.067). Likewise, leukocyte counts (p = 0.032), the inflammatory markers IL-6 (p = 0.032) and procalcitonin (p = 0.001) as well as D-dimers (p = 0.017) and INR (p<0.001) were less frequently increased in patients on ARB (Fig 5). Thus, in patients with cardiovascular comorbidities suffering from COVID-19, the association between ARB intake and reduced mortality appears to be accompanied by reduced cardiac damage and ameliorated thrombo-inflammatory responses after SARS-CoV2 infection.

**Fig 6:**
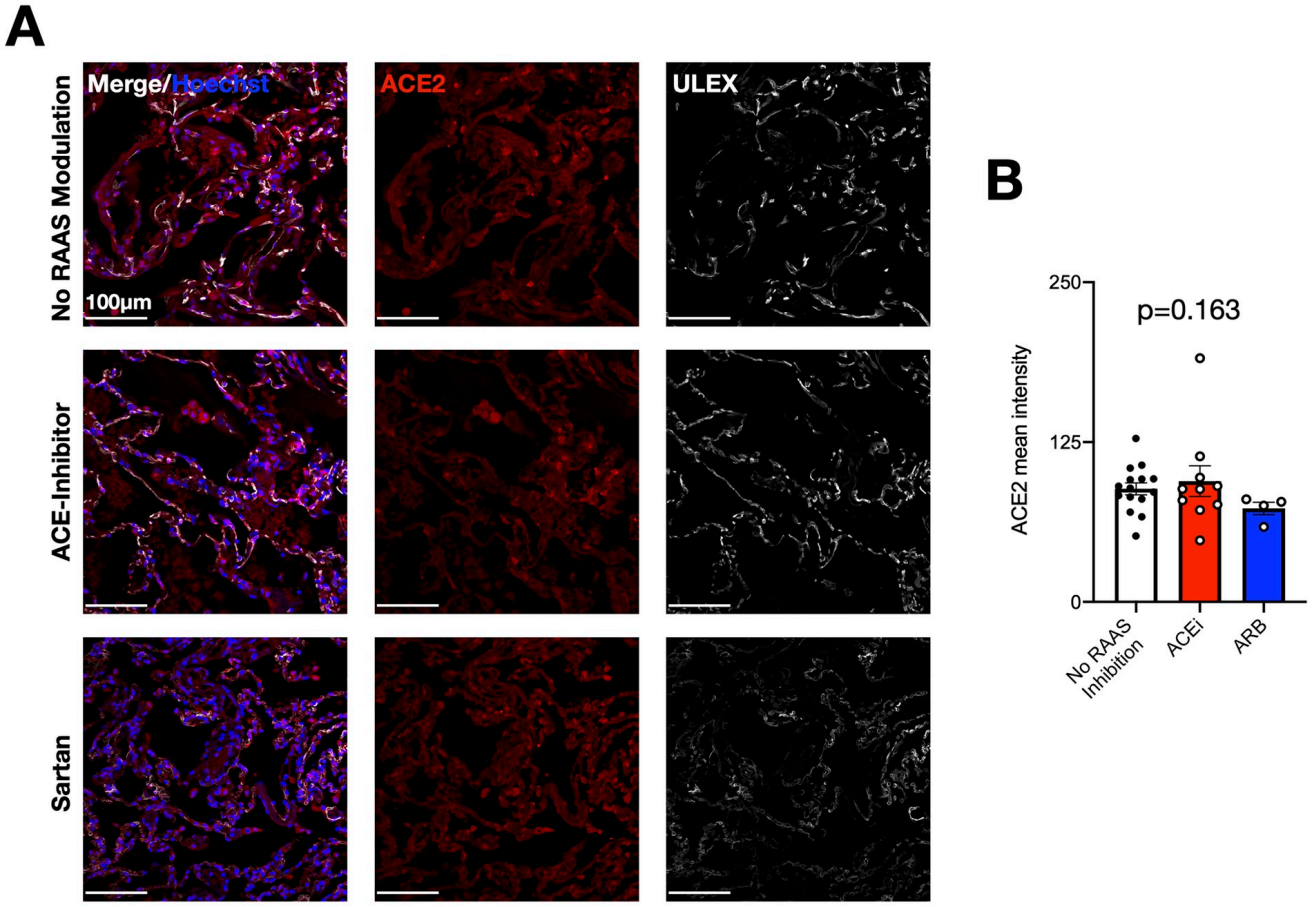
ACEi or ARB do not affect expression of ACE2 in lung epithelial cells. (A) Lung stainings of patients without RAAS modulators, with ACEi treatment and ARB medication for ACE-2 (red), Hoechst (blue) and Ulex (white). (B) Quantification of ACE-2 expression mean intensity, arbitrary units. mean±SEM; Kruskal-Wallis-test.

Finally, in order to determine whether treatment with ACEi or ARB did affect expression of the putative SARS-CoV-2 receptor ACE2 in lung epithelial cells, we investigated lung specimens from patients with or without chronic treatment by RAAS modulation. As illustrated in Fig 6, chronic pretreatment with ACEi or ARB did not increase immunohistochemically detected ACE2 protein in lung epithelial cells.

## Discussion

The present study specifically focused on patients with cardiovascular disease and hypertension, as observational studies demonstrated significantly worse outcome of these patients after contracting COVID-19 [4, 5]. By focusing on this group, we demonstrate that cardiovascular comorbidities, especially atherosclerosis and its complications as well as chronic heart failure are the predominant drivers of increased mortality in this group of patients, exceeding the risk of patients with hypertension by far. As modulation of the RAAS constitutes a first-line pharmacological treatment in patients with cardiovascular disease and hypertension [15] and the putative SARS-CoV-2 receptor ACE2 is part of the RAAS [7], it is important to exclude a potential interference of clinically used modulators of the RAAS with COVID-19 severity in patients with cardiovascular comorbidities. The results of the present study demonstrate that, while ACEi did not affect mortality after COVID-19, treatment with ARBs instead was independently associated with reduced overall mortality paralleled by mitigated cardiac damage and ameliorated thrombo-inflammatory responses after SARS-CoV-2 infection in patients with cardiovascular comorbidities.

The virus eliciting COVID-19 disease, SARS-CoV-2, utilizes the ACE2 receptor, which is highly expressed on epithelial cells in the lung, as entry for cell infection [7]. ACE2 is part of the RAAS pathway, which controls vascular tone but also regulates inflammatory and fibrotic processes [16]. In the classical RAAS-pathway, angiotensin II binds to angiotensin II type 1 receptor, which results in increased sympathetic activity, vasoconstriction, fibrosis, and inflammation. ACE2 counteracts these effects by cleaving angiotensin II into angiotensin 1–7 and angiotensin I into angiotensin 1–9, which mediate vasodilatation and suppression of inflammation via the mas receptor or the angiotensin II type 2 receptor, respectively [17]. Preclinical data described upregulation of ACE2 by either ACEi or ARBs which may on one hand exert beneficial effects by suppressing inflammation and protect cells from increased sympathetic activity [18], but on the other hand raised concerns that this would result in increased risk for infection with SARS-CoV2 and a higher risk for a complicated course of the disease. In line with other recent studies [10–12], the present results document that blockade of the RAAS by using either ACEi or ARB does not aggravate the clinical course of COVID-19, leading to death in patients with cardiovascular comorbidities. These findings are corroborated by the results of a very recent randomized protective study, which demonstrated that discontinuation of RAAS inhibition by either ACEi or ARBs did not affect the maximum severity of COVID-19 disease, yet did lead to faster and better recovery with no differences between ACEi or ARB discontinuation [19]. However, in the present study analyzing data of the LEOSS registry, most surprisingly was that ARB intake was associated with a significant and independent reduction in overall mortality of COVID-19 patients with cardiovascular disease and/or hypertension.

While earlier studies indeed suggested that pharmacological RAAS inhibition can increase ACE2 expression in organs including the heart [20–22], more recent observations questioned these studies [23]. More importantly, there is currently no solid scientific evidence that specifically ARBs do upregulate ACE2 expression [24]. Therefore, we cannot firmly conclude that the different outcome for patients on ACEi medication compared to those on ARB treatment is related to altered ACE2 expression.

ARBs specifically block the AT1 receptor and indirectly increase angiotensin II levels, which then could have additional effects by stimulating a second receptor AT2, which is insensitive to ARB but mediates anti-inflammatory effects via NFkB [25]. Indeed, our results demonstrate significantly reduced thrombo-inflammatory responses to SARS-CoV-2 infection in patients taking ARB, but not in those taking ACEi. In contrast, ACE2 expression in lung tissue was not altered in patients on chronic ACEi or ARB treatment arguing against an increased susceptibility to SARS-CoV-2 infection, which corroborates clinical observations that the risk of testing positive for SARS-CoV2 is not increased in patients taking RAAS modulators [10, 26].

As our data show that the protective effect of ARBs appears to be mainly driven by patients, who present in the uncomplicated phase of the disease at baseline, this class of drug exerts its effect most likely by limiting inflammatory disease progression after contracting the infection. This was accompanied with a strong trend towards reduced circulating levels of troponin suggestive of reduced cardiac damage especially in patients on ARB during the uncomplicated phase of COVID-19.

In a very recent small-scale study examining the interaction between inflammatory cells and epithelial cells of the nasopharynx system, ACEi intake was linked to dampened activation of the immune system in patients with COVID-19, whereas ARB treatment by itself was associated with proinflammatory features [27]. However, it should be noted that elevated inflammatory biomarkers are rather indicating severity of infection than representing the presence of a self-amplifying inflammatory circuit, which propagates tissue destruction in patients with COVID-19. In line with this, our biomarker results demonstrate lower levels of inflammation and thrombogenic activation, both parameters of disease severity [28, 29], in patients taking ARBs, who have less mortality after infection with SARS-CoV2. One potential explanation for this observation is that higher inflammatory activity at the site of infection, as described for ARBs, prevents systemic spread of the disease, which is mirrored in lower levels of systemic proinflammatory biomarkers as indicators for disease severity.

The present study has several limitations. First, by virtue of the design of the registry to anonymously capture patient data, we are not able to provide absolute values for individual biomarkers, which were divided into separate categories. In addition, we do not have sequential biomarker measurements and thus cannot comment on the potential role of dynamic alterations in the process of disease progression. Moreover, the duration of RAAS inhibition prior to contracting COVID-19 cannot be specified due to the design of the LEOSS registry. Finally, the potential mechanisms by which different RAAS modulators affect outcomes in COVID-19 disease remain unexplored and require further studies.

Our analysis specifically distinguished between ACEi and ARB and took their different modes of action into account. Importantly, the present study is the first to link inflammatory biomarker levels with the intake of either ACEi or ARBs. In summary, as the COVID-19 pandemic will remain relevant for patients with cardiovascular disease, the data presented here should encourage a randomized trial comparing ACEi and ARBs in patients with COVID-19 disease and cardiovascular comorbidities. Until such data are available, our data support current guideline recommendations not to discontinue ACEi or ARBs in patients with COVID-19.
